# Sunlight inhibits growth and induces markers of programmed cell death in *Plasmodium falciparum* in vitro

**DOI:** 10.1186/s12936-015-0867-0

**Published:** 2015-09-29

**Authors:** Dewaldt Engelbrecht, Thérèsa Louise Coetzer

**Affiliations:** Department of Molecular Medicine and Haematology, Faculty of Health Sciences, School of Pathology, Wits Medical School, Wits Research Institute for Malaria, University of the Witwatersrand, 7th floor, 7 York Road, Parktown, Johannesburg, 2193 South Africa; National Health Laboratory Service, Johannesburg, South Africa

**Keywords:** *Plasmodium falciparum*, Programmed cell death, Sunlight

## Abstract

**Background:**

*Plasmodium falciparum* is responsible for the majority of global malaria deaths. During the pathogenic blood stages of infection, a rapid increase in parasitaemia threatens the survival of the host before transmission of slow-maturing sexual parasites to the mosquito vector to continue the life cycle. Programmed cell death (PCD) may provide the parasite with the means to control its burden on the host and thereby ensure its own survival. Various environmental stress factors encountered during malaria may induce PCD in *P. falciparum*. This study is the first to characterize parasite cell death in response to natural sunlight.

**Methods:**

The 3D7 strain of *P. falciparum* was cultured in vitro in donor erythrocytes. Synchronized and mixed-stage parasitized cultures were exposed to sunlight for 1 h and compared to cultures maintained in the dark, 24 h later. Mixed-stage parasites were also subjected to a second one-hour exposure at 24 h and assessed at 48 h. Parasitaemia was measured daily by flow cytometry. Biochemical markers of cell death were assessed, including DNA fragmentation, mitochondrial membrane polarization and phosphatidylserine externalization.

**Results:**

Sunlight inhibited *P. falciparum* growth in vitro. Late-stage parasites were more severely affected than early stages. However, some late-stage parasites survived exposure to sunlight to form new rings 24 h later, as would be expected during PCD whereby only a portion of the population dies. DNA fragmentation was observed at 24 and 48 h and preceded mitochondrial hyperpolarization in mixed-stage parasites at 48 h. Mitochondrial hyperpolarization likely resulted from increased oxidative stress. Although data suggested increased phosphatidylserine externalization in mixed-stage parasites, results were not statistically significant.

**Conclusion:**

The combination of biochemical markers and the survival of some parasites, despite exposure to a lethal stimulus, support the occurrence of PCD in *P. falciparum*.

## Background

During the recurring erythrocytic stages of *Plasmodium falciparum* malaria, each mature schizont-stage parasite produces around 20 new infective merozoites [1], resulting in an exponential increase in parasite load every 48 h. Severe parasitaemia threatens the survival of the human host before transmission of the slow-maturing gametocytes to the *Anopheles* mosquito, and thus careful regulation of parasite density may be advantageous to both the parasite and its host. Several mechanisms are postulated to allow the parasite to regulate its own parasitaemia, including altering the rate of division, the efficiency of invasion and the rate of cell death [2]. Programmed cell death (PCD) may provide the parasite with the most effective means to regulate parasitaemia [2]. In multicellular organisms PCD fulfils essential roles in development, immunity and the maintenance of homeostasis [3–5], but it has also been shown in unicellular protozoa [6, 7], including *P. falciparum* (reviewed in [8]). Although the exact phenotype remains debated, a growing body of evidence suggests that *P. falciparum* exhibits one or several PCD phenotypes.

PCD may be induced by a number of environmental stress factors encountered by the parasite during malaria illness, including high parasite population density [9] and febrile episodes [10–12]. However, the effect of sunlight is almost entirely overlooked. The considerable cardiac output delivered to cutaneous circulation [13] means that at any one time a significant number of intra-erythrocytic parasites are located in the superficial blood vessels, and are thus exposed to penetrating solar radiation. This proportion may further increase as a result of vasodilation during fever paroxysms [14].

Solar radiation is a potent inducer of apoptosis in various eukaryotic cell types. Natural sunlight is composed of UV-A (320–400 nm), UV-B (280–320 nm) and UV-C (200–280 nm) radiation [15], although UV-C radiation is filtered out by the atmosphere [16, 17]. UV-B radiation directly damages nuclear DNA by causing lesions such as cyclobutane pyrimidine dimers and pyrimidine 6–4 pyrimidone photoproducts [18–20]. However, UV-B may also indirectly induce apoptosis through the generation of reactive oxygen species (ROS) that in turn triggers mitochondrial cytochrome-c release [20–22]. UV-B radiation may also cause apoptosis via other cytoplasmic or membrane targets, such as direct activation of membrane-bound death receptors (reviewed in [22]). UV-A radiation causes oxidative stress that damages and permeabilizes lipid membranes (reviewed in [23]). In murine lymphoma cells, UV-A radiation was shown to induce apoptosis in less than four hours, while the apoptotic effects of UV-B and UV-C wavelengths were delayed (reviewed in [23]).

The in vitro photosensitivity of *P. falciparum* has previously been noted in the authors’ laboratory [24], but data on *Plasmodium* spp. are otherwise limited to the effect of UV radiation on the murine host [25]. In other protists, UV-B exposure did not affect the viability of *Leishmania major* parasites either in vitro or in vivo [26] and had no impact on parasite infectivity [26, 27].

The present study is the first to investigate biochemical markers of PCD in *P. falciparum* parasites in response to natural sunlight, a physiologically relevant stress factor. It should be noted that the present study focused on the effect of natural sunlight, rather than a specific wavelength spectrum. The use of culture flasks that were not UV transparent may have prevented light in the UV spectrum from reaching parasites, whereas physiological exposure would include UV light. However, even with the exclusion of the UV spectrum, the effects observed in this study derived from natural sunlight. Exposure to sunlight caused growth inhibition and induced PCD in a sub-population of parasites. Late-stage parasites—trophozoites and schizonts—were far more affected than ring-stage parasites. Sunlight caused DNA fragmentation that preceded mitochondrial hyperpolarization, suggesting a unique form of PCD in *P. falciparum* that is not initiated by the mitochondrion. These findings provide important new information on cell death mechanisms that are utilized by the parasite to limit its population and thereby prevent premature death of the human host and thus ensure its transmission to the mosquito vector.

## Methods

### Reagents

The APO-DIRECT TUNEL kit and FITC Annexin V Apoptosis Detection Kit II were obtained from Becton–Dickinson (BD Pharmingen, San Diego, CA, USA). Thiazole orange (TO), hydroethidine (HE), 3,3′-dihexyloxacarbocyanine iodide [DiOC_6_(3)], carbonyl cyanide *m*-chlorophenylhydrazone (CCCP) were obtained from Sigma-Aldrich (St Louis, MO, USA). Albumax II was obtained from Gibco (Gran Island, NY, USA).

### Collection of human blood and ethics clearance

Human erythrocytes (RBC) were collected from healthy volunteers as whole blood into Vacutainer^®^ ACD collection tubes (Becton–Dickinson, Franklin Lakes, NJ, USA). The plasma and buffy coat were removed after centrifugation and RBC were washed with RPMI to remove residual white blood cells. Ethics clearance for the collection of blood from volunteers by venepuncture was provided by the University of the Witwatersrand Human Research Ethics Committee (Medical) under the protocol number M130569.

### *Plasmodium falciparum* culture

The 3D7 strain of *P. falciparum* was maintained according to established methods [28] with some modifications [29]. Briefly, parasites were maintained at 37 °C in malaria culture medium (RPMI 1640, 0.5 % Albumax II and 0.21 % sodium bicarbonate, supplemented with 50 mg/l gentamycin and 50 mg/l hypoxanthine) at 5 % haematocrit in RBC. Medium was changed daily. Optimal culture pH was maintained by gassing cultures daily for 60 s with a mixture of 2 % O_2_, 5 % CO_2_ and 93 % N_2_. Parasite morphology was monitored by Giemsa-stained smears and parasitaemia and staging were assessed by thiazole orange (TO) flow cytometry.

### Synchronization of parasites to ring stages

For studies involving synchronized parasites, synchronization was performed similar to an established method [30]. Briefly, *P. falciparum*-infected red blood cells (pRBC) were centrifuged for 5 min at 1000×*g* and 25 °C. The resulting cell pellet was incubated in ten volumes of 5 % D-sorbitol for 5 min at 37 °C. Centrifugation was repeated and the cell pellet was resuspended to 5 % haematocrit with medium, returned to a clean 25 sq cm culture flask and incubated at 37 °C. This method synchronized parasites to ring stages. For studies involving late-stage parasites, pRBC cultures were allowed to mature for 20–24 h.

### Exposure to natural sunlight

pRBC were seeded as 5 ml cultures in 25 sq cm, optically clear, sealed tissue culture flasks. Sunlight exposure was performed for one hour in the middle of the day (between 10.00 and 14.00 h) on clear, sunny days in Johannesburg, South Africa, with an elevation of 1753 m above sea level. Culture flasks were maintained at 37 °C by a Lauda B Circulator (temperature uniformity: ±0.01 °C) in a Lauda MA6 water bath (Lauda-Brinkmann, Delran, NJ, USA) throughout exposure. Temperature consistency was additionally monitored by a mercury-in-glass thermometer. Previous control experiments confirmed that culture medium inside the flasks exposed to sunlight in this manner remained constant at 37 °C. Control cultures were covered with thick cardboard to prevent exposure to sunlight, while experimental cultures were left uncovered in direct sunlight. Following exposure, all cultures were returned to the dark in an incubator at 37 °C.

### Flow cytometry

Flow cytometric analyses were performed on a Beckman Coulter Gallios flow cytometer (Beckman Coulter Inc, Miami, FL, USA). Excitation for all assays was by 488 nm blue laser. Emission was detected with the use of 545/40BP (525 ± 20 nm, FL1) and, where indicated, 575/30BP (575 ± 15 nm, FL2) filters. Optical alignment was monitored daily with Beckman Coulter Flow Check Pro fluorospheres (Beckman Coulter Inc, Brea, CA, USA). Post-acquisition analyses were performed with Beckman Coulter Kaluza (v1.1) software.

### TO flow cytometry for parasitaemia

Parasitaemia was measured daily by flow cytometry with the DNA-binding dye TO, similar to a previous method [31]. Whole culture samples (10 µl) were diluted 100-fold to 1 ml in Sorenson’s phosphate buffer (47 mM Na_2_HPO_4_, 20 mM KH_2_PO_4_, pH 7.2) with 1 µM TO final concentration (diluted from a 10 mM stock in methanol) and incubated at room temperature in the dark for 20 min. Stained cells were analysed by flow cytometry within 1 hour. Erythrocytes were gated on a forward- versus side-scatter dot plot and analysed on a FL1 integral (log) histogram, with regions for uninfected, ring-infected and trophozoite- or schizont-infected erythrocytes delineated. Regions had previously been confirmed by microscopy of Giemsa-stained smears of synchronized cultures. Approximately 50,000 events in the erythrocyte gate were counted.

### TUNEL assay for DNA fragmentation

The terminal deoxynucleotidyltransferase (TdT)-mediated nick end labelling (TUNEL) assay was performed according to manufacturer’s recommendations, with modifications similar to a previous study [11]. Briefly, pRBC were pelleted and fixed on ice for 60–90 min in 4 % formaldehyde/phosphate-buffered saline (PBS: 10 mM Na_2_HPO_4_, 1.5 mM KH_2_PO_4_, 137 mMNaCl, 2.7 mMKCl, pH 7.4), followed by permeabilization with 0.1 % tri-sodium citrate (w/v) and 0.1 % Triton X-100 (v/v) in PBS for 3 min on ice. Labelling with DNA-staining solution (including TdT enzyme and FITC-dUTP) was performed according to manufacturer’s recommendations for 60–90 min at 37 °C, followed by staining with propidium iodide (PI) for 30 min at room temperature. Labelled cells were analysed by flow cytometry within three hours. PI-positive parasites were acquired on a FL2 time-of-flight (lin) versus FL2 integral (lin) dot plot, with gated parasites analysed on a FL1 integral (log) histogram for DNA fragmentation, measured as FITC-dUTP fluorescence. At least 10,000 PI-positive events were counted. DNase-treated, non-treated and unlabelled parasites were used as positive, negative and staining controls, respectively.

### DiOC_6_(3) flow cytometry for mitochondrial transmembrane potential

*Plasmodium falciparum* whole culture samples (50 µl) were diluted to 1 ml in PBS containing 10 nM DiOC_6_(3) (diluted from a 100 mM stock in DMSO) and 50 µM hydroethidine HE (diluted from a 10 mM stock in DMSO) and incubated at 37 °C for 45 min in the dark. Following incubation, cells were washed and suspended in 1 ml PBS and analysed immediately by flow cytometry. Erythrocytes were gated on a forward- *versus* side-scatter dot blot. HE-positive pRBC counted on a FL2 integral (log) histogram were analysed for DiOC_6_(3) fluorescence on a FL1 integral (log) histogram. At least 10,000 HE-positive events were counted. Positive controls were treated with 200 nM CCCP for one hour before staining. Unstained cells, cells stained with only HE or DiOC_6_(3) and non-parasitized erythrocytes were used as staining controls.

### Annexin V-FITC for PS externalization

*Plasmodium falciparum* culture samples (20 µl) were diluted 50-fold to 1 ml in PBS and stained with a final concentration of 50 µM HE (diluted from a 10 mM stock in DMSO) in PBS for 15 min at 37 °C in the dark. Cells were then pelleted, suspended to 100 µl in 1X annexin-binding buffer (provided with the kit) and stained with annexin V-FITC for 15 min in the dark according to manufacturer’s recommendations with modifications similar to a previous study [32]. Stained cells were diluted to 1 ml in 1X annexin-binding buffer and analysed by flow cytometry within 1 h. Erythrocytes were gated on a forward- *versus* side-scatter dot blot and pRBC were discriminated on a FL 2 integral (log) histogram for HE fluorescence. Gated pRBC were analysed for annexin V-FITC fluorescence on a FL1 integral (log) histogram. At least 50,000 events in the erythrocyte gate were counted. pRBC treated with recombinant annexin V (BD Pharmingen, San Diego, CA, USA) before staining were used as a negative control and unstained parasite cultures and parasite cultures stained with only HE or annexin V-FITC were used as staining controls.

### Statistical analysis

Bar graphs were compiled using GraphPad Prism 5, with raw data values exported from analyses by Beckman Coulter Kaluza (v.1.1) software. Student’s unpaired *t* tests were performed with Microsoft Office Excel 2010 to test for significance between treated and control groups. Data distributions were not tested for normality.

## Results

### Sunlight inhibited *Plasmodium falciparum* growth in vitro

**Fig. 1:**
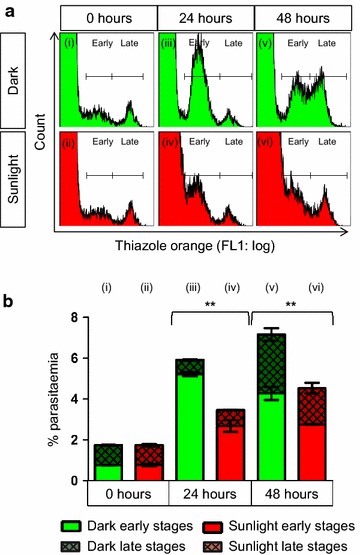
Mixed-stage *Plasmodium falciparum* growth decreased after exposure to sunlight in vitro. Flow cytometry histograms (**a**) and statistical analyses (**b**) showed that late-stage parasites (*Late*) were more affected by exposure to solar radiation than early stage parasites (*Early*). When compared to experimental cultures exposed to sunlight for 1 h (*Sunlight*), control cultures (*Dark*) showed a large population of new early-stage parasites (*iii*), whereas the formation of early stage parasites was significantly reduced in experimental cultures (*iv*) indicating that late-stage parasites exposed to sunlight produced fewer new rings. Statistical comparisons were made between total parasitaemia values and were significant at P < 0.01 (*double asterisk*). For all comparisons, n = 2

**Fig. 2:**
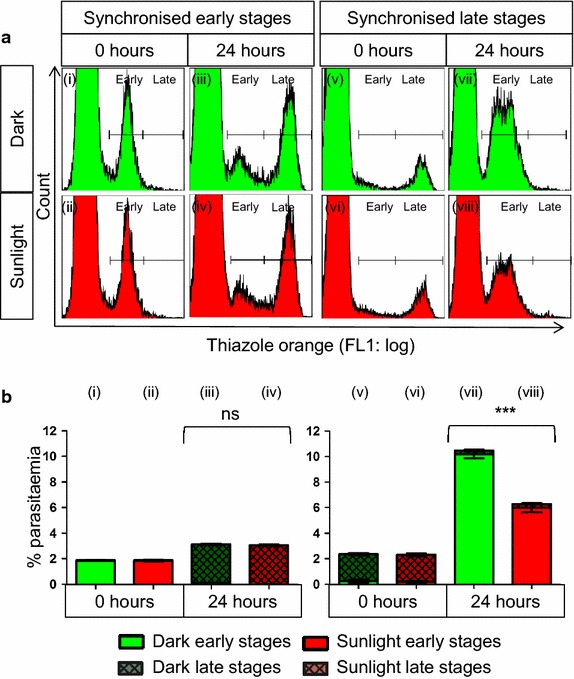
Sunlight decreased the growth of late-stage *Plasmodium falciparum*, but early stages were unaffected. Flow cytometry histograms (**a**) and statistical analyses (**b**) showed that early-stage parasites (*i* **–** *iv*) were unaffected by exposure to solar radiation, whereas late-stage parasites (*v* **–** *viii*) were significantly affected. Synchronized early-stage parasites showed no difference in development between cultures maintained in the dark (*Dark*) and those exposed to sunlight for one hour (*Sunlight*), while synchronized late-stage parasites showed significantly reduced growth (*viii*). Statistical comparisons were made between total parasitaemia values and were not significant (ns) or significant at P < 0.001 (*triple asterisk*). For all comparisons, n = 4

Mixed and synchronized parasite cultures were exposed to one hour of sunlight and assessed for growth 24 h later. Mixed-stage cultures were exposed to another 1 h of sunlight at 24 h and assessed again 24 h later, at 48 h. Sunlight decreased the in vitro growth of both mixed (Fig. 1) and synchronized (Fig. 2) parasite cultures.

Early-stage parasites were unaffected by sunlight. Twenty-four hours after a single exposure, ring stage parasites in both mixed stage (Fig. 1aii, bii) and synchronized (Fig. 2aii, bii) parasites progressed to trophozoite stages (Figs. 1aiv, biv, 2aiv, biv, respectively) similar to control cultures maintained in the dark (Figs. 1aiii, biii, 2aiii, biii, respectively).

In contrast, sunlight decreased the growth of late stage parasites in both mixed (Fig. 1) and synchronized (Fig. 2) cultures. Fewer new rings were formed (Figs. 1aiv, biv, 2aiii, bviii) from late-stage parasites exposed to sunlight (Figs. 1ai, bi, 2avi, bvi) than from corresponding cultures maintained in the dark. However, growth inhibition was only partial, as evidenced by increased parasitaemia between 0 and 24 h, albeit less than in control cultures.

In mixed-stage cultures, a decrease in both early and late stage parasites was seen at 48 h (Fig. 1avi, bvi vs. Av and Bv), after a second dose of sunlight was delivered at 24 h. A decrease in late-stage parasites, compared to control cultures at 48 h, is attributed to the initial exposure of trophozoites at 0 h causing fewer rings at 24 h and correspondingly fewer late stage parasites at 48 h. As sunlight had no effect on the growth of early-stage parasites, only mixed-stage parasites and synchronized late-stage parasites were used for cell death assays.

### Exposure to sunlight caused DNA fragmentation in *Plasmodium falciparum*

**Fig. 3:**
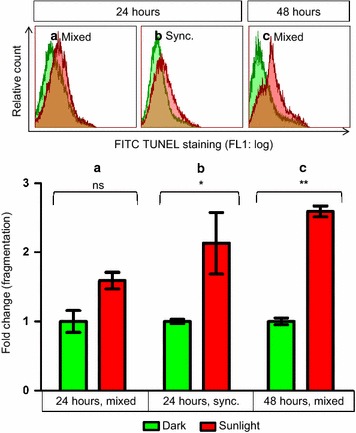
Exposure to sunlight caused DNA fragmentation in *Plasmodium falciparum* in vitro. Although DNA fragmentation was not observed in mixed-stage parasites after a single exposure to sunlight (**a**), synchronized late-stage parasites did show DNA fragmentation at 24 h (**b**). DNA fragmentation was also seen in mixed-stage parasites at 48 h, after two exposures to sunlight (**c**). Statistical comparisons were not significant (ns), or significant at P < 0.05 (*asterisk*) or at P < 0.01 (*double asterisk*). n = 2 for (**a**) and (**b**); n = 4 for (**c**)

DNA fragmentation was quantified from fixed and isolated parasites by the flow cytometric TUNEL assay, 24 h after every sunlight exposure. DNA fragmentation was seen at 24 h in synchronized late stage parasites (Fig. 3b) and at 48 h in mixed-stage parasites (Fig. 3c).

Mixed-stage parasites showed suggestive but insignificant DNA fragmentation at 24 h (Fig. 3a), likely due to the negating effect of early-stage parasites that were unaffected by sunlight that reduced the fragmentation of the population as a whole. Therefore, it seems likely that late-stage parasites in mixed cultures exhibited DNA fragmentation in the same manner as synchronized late-stage parasites did.

### Exposure to sunlight caused mitochondrial hyperpolarization in mixed-stage parasites

**Fig. 4:**
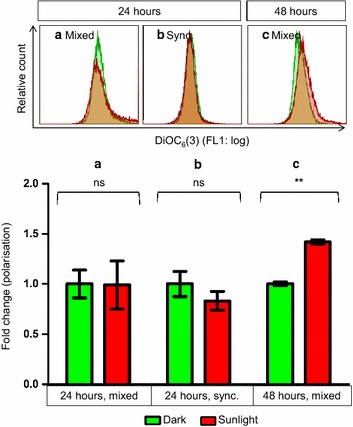
Sunlight caused changes in mitochondrial polarization in *Plasmodium falciparum* only after 48 h. No change was seen in the mitochondrial polarization of mixed-stage parasites (**a**) or synchronized late-stage parasites (**b**) exposed to sunlight for 1 h and maintained in the dark for the remainder of 24 h, compared to control parasites kept in the dark. After a second exposure to solar radiation, mixed-stage parasites showed an apparent increase in mitochondrial polarization at 48 h, compared to parasites maintained in the dark (**c**). Statistical comparisons were not significant (ns) or significant at P < 0.01 (*double asterisk*). n = 2 for (**a**) and (**b**); n = 4 for (**c**)

Mixed-stage parasites showed increased DiOC_6_(3) fluorescence, suggesting mitochondrial hyperpolarization, the day following a second exposure to sunlight (Fig. 4c). No mitochondrial dysregulation was observed in synchronized late-stage parasites or in mixed-stage parasites 24 h after the first exposure to sunlight (Fig. 4a, b). DNA fragmentation therefore preceded mitochondrial dysregulation, with DNA fragmentation observed in late stage parasites at 24 h, and mitochondrial hyperpolarization only observed at 48 h in mixed-stage parasites. Mitochondrial hyperpolarization was likely due to increased reactive oxygen species (ROS) [10, 24], but it is not clear whether there is any causative relationship between DNA fragmentation and the appearance of mitochondrial hyperpolarization.

### Exposure to sunlight caused no change in phosphatidylserine externalization

**Fig. 5:**
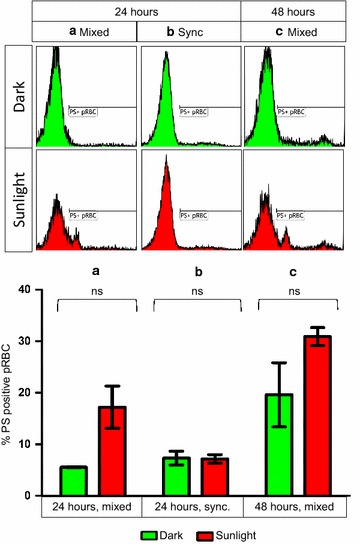
No significant change was seen in the phosphatidylserine externalization of parasitized erythrocytes after exposure sunlight. Flow cytometry histograms (*upper panels*) showed an apparent increase in PS externalization in mixed-stage pRBC at both 24 (**a**) and 48 (**c**) hours after one and two exposures to sunlight, respectively. However, statistical analyses of *bar graphs* (*bottom panels*) showed no significant differences (ns). Synchronized late-stage pRBC exposed to solar radiation for 1 h and maintained in the dark for the remainder of 24 h also showed no change in PS externalization, compared to control cultures maintained in the dark (**b**). n = 2 for (**a**) and (**b**); n = 4 for (**c**)

Flow cytometry data showed increased PS externalization in mixed-stage pRBC on the day following both the first (Fig. 5a) and second exposure (Fig. 5c) to natural sunlight, but the difference was not statistically significant. No increase in PS externalization was observed in synchronized late-stage pRBC exposed to sunlight (Fig. 5b).

## Discussion

### Shedding light on a killer: sunlight inhibits *Plasmodium falciparum* growth

Since ancient times, humans have linked sunlight and malaria and one of the myths was that staying out in the sun would cause malaria [33]. However, sun worship in early cultures may have in fact conferred a survival advantage to its practitioners, by reducing the burden of disease [Mendelow and Coetzer, unpublished observations]. The data from this study showed that exposure to sunlight inhibited the in vitro growth of intra-erythrocytic *P. falciparum.* The effect of sunlight was tested in both mixed and synchronous parasites. In vivo malaria often involves multiple, asynchronous infections [34]. Therefore, the use of mixed parasite populations with multiple sunlight exposures was essential to determine the effect of sunlight in conditions that mimic in vivo disease. However, the use of synchronous parasites with a single exposure allows further narrowing of the effects of sunlight on different asexual developmental stages. Early-stage parasites were resistant to damage caused by sunlight and although late-stage parasites were more severely affected, some late-stage parasites survived sunlight exposure. These may have been early trophozoites at the time of exposure, rather than late trophozoites or schizonts. The conservation of some parasites, despite exposure to a lethal stress stimulus, supports the occurrence of PCD, which requires a portion of the population to survive. It is not clear whether decreased parasite growth was due to fewer schizonts being produced or if some of the newly released merozoites were non-viable and hence could not invade new erythrocytes. Another possibility for reduced parasite growth that also conserves a portion of the population may involve some quorum sensing-like mechanism, with parasite-derived signalling molecules resulting in the induction of PCD. However, such a mechanism has not been identified in *P. falciparum* and furthermore it is not clear why only a portion of the parasite population would react to such signalling molecules (reviewed in [8]). It seems more likely that the conservation of some parasites relates to the specific asexual developmental stage of those parasites. The details of this mechanism require further elucidation.

To further investigate the decline in parasitaemia after exposure to sunlight, biochemical markers of cell death were evaluated. DNA fragmentation is one of the characteristics of PCD and was observed in late-stage parasites, confirming that the reduced growth resulted from the death of some parasites, rather than the reduced reproduction of the population as a whole. DNA fragmentation has been widely reported in *P. falciparum* in response to anti-malarial drugs [2, 35–38], heat stress [10, 11], high parasite density [9], bilirubin [39], etoposide [36], and staurosporine [37]. Some studies have attributed the occurrence of DNA fragmentation, without other biochemical markers of cell death, to apoptosis in *P. falciparum* [2, 11, 36], although it has also been attributed to an undefined pathway [40].

The effect of sunlight on another key element of PCD, the polarization of the mitochondrial membrane, was also investigated. Mitochondrial hyperpolarization was observed in mixed-stage parasites the day following a second sunlight exposure. Although mitochondrial depolarization, resulting from permeabilization of the outer mitochondrial membrane, is expected during apoptosis [41–46], both mitochondrial depolarization and hyperpolarization may form part of an apoptosis response in other protozoa [47–49]. The asexual stages of *P. falciparum* contain only a single mitochondrion that is chiefly involved in pyrimidine synthesis [50]; however, previous studies have shown the importance of mitochondria in parasite programmed cell death [51]. It should also be noted that transient hyperpolarization in metazoans may occur as an early checkpoint in deciding cell fate [48]; however, hyperpolarization in this case was not observed immediately after exposure to sunlight, but only a day after the second exposure and two days after the first. Mitochondrial hyperpolarization was likely the result of increased accumulation of reactive oxygen species (ROS), which has previously been suggested to be the cause of parasite death after sunlight exposure [24]. The previous study showed that *P. falciparum* growth is reduced and the developmental cycle of parasites delayed by 30-min daily exposures to natural sunlight for 4 days. The observed effects were ascribed to photo-activation of ROS, possibly free erythrocyte protoporphyrin [24].

The externalization of PS in pRBC was evaluated as a third marker of PCD. PS externalization has previously been noted to result from parasite maturation or stress [32, 52]. Although results suggested increased PS externalization in mixed-stage cultures, the results were not statistically significant. The absence of increased PS externalization, particularly in late-stage pRBC, supports the notion of a unique PCD pathway in *P. falciparum*.

These combined data therefore showed that sunlight exposure induced DNA fragmentation followed by mitochondrial hyperpolarization in the absence of PS externalization. This unusual manifestation of biochemical markers suggests a cell death phenotype that does not entirely fit with any metazoan PCD models and might be unique to *P. falciparum*.

### Sunlight and heat stress: cooperative effect

*Plasmodium falciparum* utilizes a unique method of erythrocyte modification to allow adhesion of late-stage asexual and immature sexual parasites to endothelium, thereby sparing parasites from splenic clearance [1]. Heat stress, such as febrile episodes characteristic of malaria [1], increases cardiac output to cutaneous circulation to as much as 60 % of total vascular conductance [13] and further increases the cyto-adherence of mature *P. falciparum*-infected RBC [53]. Therefore, a large number of late-stage parasites would be present in the microvasculature near the skin and thus be exposed to sunlight. Heat stress reduces *P. falciparum* numbers in vitro [10–12, 34, 54, 55] and has been suggested to cause PCD in the parasite [10, 11]. Sunlight and heat stress may therefore have a cooperative effect in parasite clearance.

### Sunlight and heat stress: different stress, different phenotype

In contrast to the effect of sunlight on late-stage pRBC, PCD induced by heat stress was characterized by mitochondrial hyperpolarization and PS externalization without any DNA fragmentation [10]. Interestingly, heat stress also induced PCD in some early stage parasites that exhibited DNA fragmentation and mitochondrial depolarization [10], whereas sunlight did not affect ring stages. Therefore, *P. falciparum* shows unique combinations of cell death markers that differ depending on the type of stress.

Early-stage parasites appear to be well adapted to surviving lethal stress stimuli. In vivo, the rupture of pRBC and the egress of merozoites are responsible for the onset of fever, but the merozoites would have re-invaded and be present as ring-stage parasites by the time heat stress occurs and will thus not be affected [10, 56]. In the case of sunlight, early-stage parasites are exposed as a result of continuous circulation, but since they do not sequester as late-stage parasites do, they presumably receive less sunlight than late-stage parasites.

## Conclusion

In vitro exposure to natural sunlight reduced the growth of *P. falciparum* due to the death of a portion of the parasite population. The occurrence of DNA fragmentation prior to mitochondrial depolarization suggests a cell death mechanism that is not initiated by the mitochondrion. This combination of biochemical markers may offer clues to a unique phenotype of PCD in *P. falciparum*. Although conflicting data concerning the phenotype of PCD in *P. falciparum* might be attributed to the use of various strains and cell death markers, it is also apparent that *P. falciparum* may vary its response to different potentially fatal stimuli. However, whether this should be attributed to diverse underlying mechanisms or simply different facets of the same pathway remains unclear.

## Abbreviations

CCCP: carbonyl cyanide *m*-chlorophenylhydrazone
DiOC_6_(3): 3,3′-dihexyloxacarbocyanine iodide
HE: hydroethidine
PBS: phosphate-buffered saline
PCD: programmed cell death
PI: propidium iodide
pRBC: *Plasmodium falciparum*-infected red blood cells
PS: phosphatidylserine
ROS: reactive oxygen species
TdT: terminal deoxynucleotidyltransferase
TO: thiazole orange
TUNEL: TdT-mediated nick end labelling
